# Biomonitoring of airborne metals using tree leaves: Protocol for biomonitor selection and spatial trend

**DOI:** 10.1016/j.mex.2019.07.019

**Published:** 2019-07-20

**Authors:** Yaghoub Hajizadeh, Mehdi Mokhtari, Maryam Faraji, Ali Abdolahnejad, Amir Mohammadi

**Affiliations:** a Environment Research Center, Research Institute for Primordial Prevention of Non-Communicable Disease, Isfahan University of Medical Sciences, Isfahan, Iran; b Environmental Science and Technology Research Center, Department of Environmental Health Engineering, Shahid Sadoughi University of Medical Sciences, Yazd, Iran; c Environmental Health Engineering Research Center, Kerman University of Medical Sciences, Kerman, Iran; d Department of Environmental Health, School of Public Health, Kerman University of Medical Sciences, Kerman, Iran; e Department of Public Health, Maragheh University of Medical sciences, Maragheh, Iran

**Keywords:** Biomonitoring and mapping of airborne metals using tree leave, Biomonitoring, Metals, GIS

## Abstract

In northwest of Iran, airborne particulate matter originated from drying Urmia Lake is threaten the health of surrounding communities due to salt particles and heavy metals. This study aimed to use leave of local trees for biomonitoring of toxic metals and to evaluate tolerance of the trees against air pollution due to greenbelt development. Leaf samples were taken from four dominant tree species including *Vitis vinifera, Juglans regia, Ulmus umbraculifera* and *Popolus alba* in two radial distances (5 and 10 km) around the Urmia Lake in 32 sampling sites. The concentration of Cd, Pb, Ni, As, Cu, Zn and Na in the leaves were extracted according to method 3050B defined by United States Environmental Protection Agency (USEPA) and analyzed by ICP-AES technique. According to the levels of air pollution tolerance index (APTI), *Popolus. alba* was classified as more sensitive and *Vitis. vinifera* as moderately tolerant. The accumulation/existence of metals in the leaves can be arranged as follows: Na > Zn > Cu > Ni > Pb > As > Cd. Our findings showed that *Popolus. alba* can be applied as a local biomonitor and *Vitis. vinifera* can be used as a good sink of air pollutants for greenbelt development around the drying Urmia Lake.

• The results show that APTI is a suitable index for selection of tree species as biomonitor and green belt development.
• Determination of metal concentration level in local tree leaves is suggested as a good tool for mapping of airborne metal.
• The local trees can be suitable for development of greenbelt in order to improve air quality, and *also* for biomonitoring of air pollution.

## Graphical abstract

**Figure:**
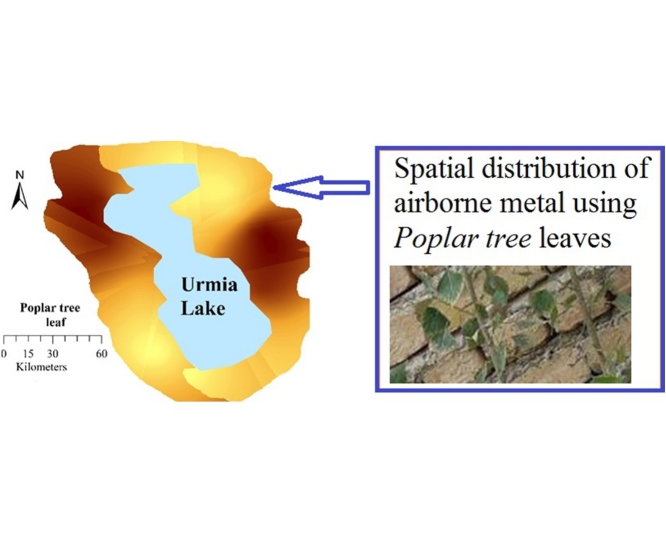

**Specification Table**

**Table:** 

Subject Area:	Environmental Science
More specific subject area:	Air pollution biomonitoring
Protocol name:	Biomonitoring and mapping of airborne metals using tree leave
Reagents/tools:	inductively coupled plasma-atomic emission spectrometry (ICP-AES, Model: Arcous)
Experimental design:	After sampling and analysis, air pollution tolerance index (APTI) of four tree leave species was calculated using a mathematical formula by replacing values of ascorbic acid content, leaf-extract pH, total chlorophyll content and relative water content (RWC) [1]. Spatial trend of metals detected by ICP-AES was determined using ArcGIS software, version10.1. The Kriging interpolation technique was applied to generate independent raster layers for metal elements [2].
Trial registration:	
Ethics:	Not required

**Value of the Protocol**

**Table:** 

• Protocol is easy, efficient and low cost • It can be used for selection of suit tree species by APTI index for biomonitoring • It can be used to evaluate ecological risk assessment and spatial trend of airborne metals

## Description of protocol

Airborne particulate matter (PM) with organic and inorganic compounds are the noticeable factors of air pollution emitted primarily from soil, fuel combustion and industrial activities [3,4]. PM may possibly contain toxic elements that can be transferred by natural processes to the hydrosphere and biota, traveling more distances from the main source [5]. Origin of PM is not only related to anthropogenic activity, but also natural phenomena such as dust storm in the deserts, and dried bed of rivers, ponds or lakes [[6], [7], [8]].

### Study area and sampling sites

**Fig. 1:**
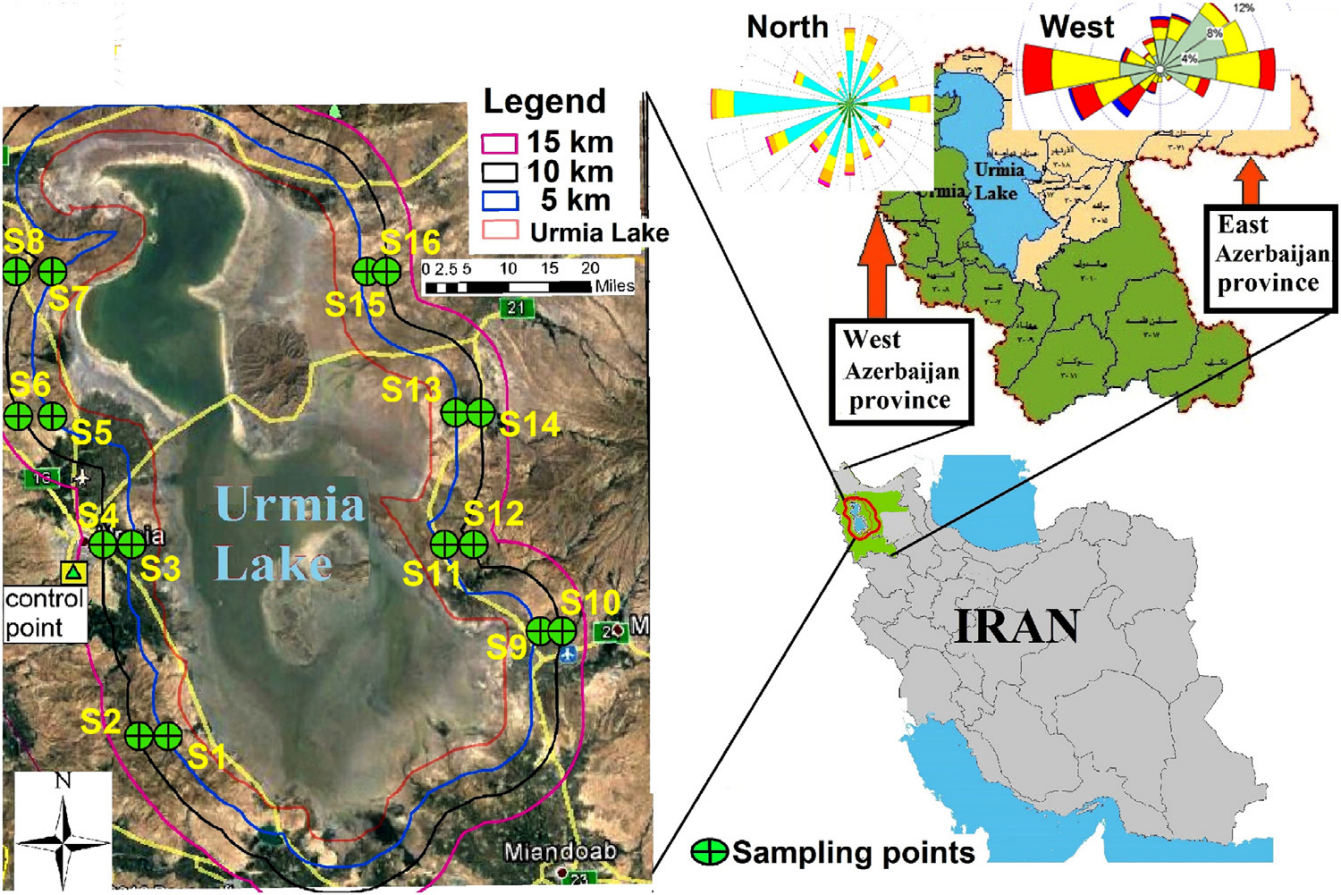
Study area.

The study area occupies 4000 km^2^ is located around the Urmia Lake (latitudes of 38°44'−38°45′N and the longitudes of 37°45'−37°46′E) northwest of Iran. The climate of this area is mainly influenced by the rainy winds of the Atlantic Ocean and Mediterranean. Also, the prevailing wind direction at the sampling points was often from Urmia Lake to east and west area [9]. Leaf samples were collected in two radial distances (5 and 10 km) around the Urmia Lake (Fig. 1). Number of sampling areas were 32 points. A control point was considered in the unpolluted lands between Sir Mountain and Shaharchi River which is protected by Urmia environmental protection office (Fig. 1).

### Tree species sampling

In the study area, four dominant tree species including *Vitis vinifera, Juglans regia, Ulmus umbraculifera and Popolus alba* were identified. Samples of the tree leaves were collected from April to September 2017, in spring and summer. Total of 192 samples were collected from 32 sites. The trees had approximately same trunk diameter and age (about 10–15 years old) and a height of 1.5–2 m above the ground. According to the VDI-Guideline 3975, part 11 [10], sampling was done triplicate from each tree species at each site and their average values were reported. Leaf samples of 70 g was taken by gloves from each tree and protect in plastic box for extraction and analysis. After sampling, leaves were immediately located in a sealed plastic bag and kept in ice-box and transferred to the lab for physicochemical analysis.

### Air pollution tolerance index (APTI)

The APTI of samples from four species, *Vitis vinifera, Juglans regia, Ulmus umbraculifera and Popolus alba,* was calculated using a mathematical formula by replacing values of ascorbic acid content, leaf-extract pH, total chlorophyll content and relative water content (RWC) [1]. The APTI formula is given in Eq. (1):

(1) *APTI=[A (T + P) +R]/10*

where, A is the ascorbic acid value of fresh leaf (mg/g); T is the total chlorophyll value of fresh leaf (mg/g), P is pH of extracted leaves and R is the relative water content (%). Measurement of ascorbic acid and total chlorophylls (a and b) value was done by spectrophotometric method [11,12]. Also, for determination of pH in samples, leaf samples were homogenized with crushing of 0.5 g leaf in 50 mL deionized water, then homogen mixture was centrifuged and supernatant was taken for measurement of pH with a digital pH meter [13]. RWC of leaves was determined by drying the previously weighted leaves in the oven at 105 °C for 3 h weighing out after that and calculating as Eq. (2):

(2) $$RWC=\frac{(FW-DW)*100}{TW-DW}$$

where FW is weight of fresh leaves, DW is weight of dried leaves in 105 °C for 3 h and TW is weight of the leaves turgid by immersion in deionized water overnight [14]. Based on APTI, three degrees of tolerance were categorized: sensitive (S) for APTI < 11, intermediate (I) for 12 < APTI < 16 and tolerant (T) for APTI > 17 [15].

### Sample preparation and metals analysis

For determination of metal elements, first 50 g of each fresh leaf samples from selected biomonitor was consecutively washed with tap water and rinsed tree times with deionized water to remove any impurity. Then, cleaned leaves were dried in the oven at 80 °C for 72 h and grinded to covert fine powder. For extraction of heavy metals, according to USEPA method 3050B, 1 g of each dried mixed sample was warmed (2 h at 50 °C) into a solution consist of HNO_3_ 70%, HCl 70% and HF 40% with ratio of 9:3:1 and then mineralized using a Teflon high-pressure digestion vial (Applied Plastics Technology, Inc., Bristol, USA) in 170 °C for 4 h. After digestion, samples were cooled and 2 mL H_2_O_2_ 30% and 3 mL concentrated HNO_3_ added to them. This solution was diluted to 50 mL with Milli-Q water and then filtered (a micro-porous membrane with the pore size of 0.45 μm) into a PET bottle. For quality control, a blank digest solution was also run as described above. Finally, elements including Fe, Cd, Cu, Ni, Pb, Zn, Na as metals and arsenic as a metalloid were detected by inductively coupled plasma-atomic emission spectrometry (ICP-AES, Model: Arcous, German), acceding to USEPA Method 200.7 [16].

### Spatial distribution of the metal elements

Spatial distribution of the heavy metals and sodium in the leaves of the trees around Urmia Lake was drawn using ArcGIS software, version10.1. The Kriging interpolation technique was applied to generate independent raster layers for metal elements. Then, the raster calculator function was used to overlay each layer to make maps of seasonal averages. The parts with high and low metal contamination were highlighted by stretch method and categorized with color signifying method. Heavy metals and sodium concentrations were higher in dark-brown regions than that in the light-brown regions [9,17].

In recent years, growing drought process of the saline Urmia Lake resulted in the emission of fine particles from the Lake dried bed to the atmosphere and thereby spreading the pollutants toward the surrounding rural, urban and agricultural lands. This dramatic phenomenon raised the concerns of communities due to the adverse health effects of air and land pollution.

### Air pollution tolerance index (APTI) of the tree species

The mean pH of leaves-extracts in the all sampling sites was obtained in the range of 6.28 ± 0.36–6.48 ± 0.64.

The mean of total chlorophyll (TCh) content (mg/g) for leaves of *Vitis vinifera, Juglans regia, Ulmus umbraculifera and Popolus alba* trees was measured at 8 ± 3.40, 5 ± 2.07, 4.3 ± 2.69 and 3.8 ± 1.66, respectively (Table 1).

**Table 1:** Mean values of air pollution tolerance index (APTI) and its related parametrs for tree species study (n = 192).

APTI parameters	tree species			
	*V. vinifera*	*J. regia*	*U. umbraculifera*	*P. alba*
pH of leaf extract	6.48 ± 0.64	6.35 ± 0.84	6.29 ± 0.40	6.28 ± 0.36
Total chlorophyll content (mg/kg)	8 ± 3.40	5 ± 2.07	4 ± 2.69	4 ± 1.66
Ascorbic acid content (mg/kg)	4 ± 2.01	3 ± 0.84	2 ± 1.23	2 ± 0.59
Relative Water Content (%)	75 ± 6.48	58 ± 11.00	51 ± 14.16	49 ± 7.25
APTI	12.57 ± 3.83	8.85 ± 1.55	7.29 ± 2.84	6.71 ± 0.99
Assessment of tree species	Moderate	Sensitive	Sensitive	Sensitive

Mean ± Standard deviation.

The mean values of ascorbic acid for *V. vinifera, J.regia, U. umbraculifera and P. alba* leaves were obtained at 4.27 ± 2.01, 3.02 ± 0.84, 2.35 ± 1.23 and 2.08 ± 0.59 mg/g, respectively.

The means of RWC for *V. vinifera, J.regia, U. umbraculifera and P. alba* leaves were obtained at 75 ± 6, 58 ± 11, 51 ± 14 and 49 ± 7%, respectively.

The mean APTI values of the tree species are given in Table 1. The maximum mean APTI value was attained for *V. vinifera* (12.57 ± 3.83) and that’s minimum value was recorded for *P. alba* (6.71 ± 0.99). Moreover, there was significant difference between the APTI values of the four tree species (P < 0.05). Based on the results in Table 1, *V. vinifera* was classified within the moderate tolerance grade and *J. regia, U. umbraculifera and P. alba were* assessed as sensitive plants. Trees with high APTI values are tolerant to pollution and can be recommended for greenbelt development as sinks of air pollutants [18]. Sensitive species with low APTI has been suggested to use as bioindicators of air pollutants [19]. Therefore, in the present study for greenbelt planning around the Urmia Lake *V. vinifera was* suggested as air pollutants receptor, and *P. alba* with lowest APTI was introduced as a good phytomonitor of air pollutants. In really Trees with high APTI value are for green belt development and sink of air pollutants. Also, trees with low values of APTI could be suitable for bioandicator.

### Heavy metals and sodium concentrations in the leaves

The concentrations of heavy metals and sodium in the trees leaves and their descriptive analysis are given in Table 2. Significant difference (P < 0.05) was found between four tree species for As, Cu, Cd, Ni, Pb, Zn and Na concentrations. Between the studied heavy metals, Zn had the maximum values and the highest value was belonged to *V. vinifera* with a mean concentration of 24.82 mg/kg. The ranks of heavy metals concentration followed the order of: Zn > Cu > Ni > Pb > As > Cd. Similar studies have reported the highest concentration of Zn between heavy metals in tree leaves [20,21] which is in agreement with our finding. This can be attributed to the role of Zn in the biosynthesis of enzymes, auxins, and some proteins in plant as micronutrient [22].

**Table 2:** Statistical descriptive of metal elements in leaves for studied tree species (n = 192).

Metal elements (mg/kg)		Tree species			
		*V. vinifera*	*J. regia*	*U. umbraculifera*	*P. alba*
As	Mean	0.23	0.49	0.15	0.15
	Std. deviation	0.12	0.24	0.09	0.09
	Range	0.05–0.42	1.18–0.23	0.05–0.32	0.05–0.30
As in control point		0.05	0.09	0.10	0.06
Cd	Mean	0.03	0.03	0.08	0.07
	Std. deviation	0.01	0.01	0.10	0.08
	Range	0.02–0.05	0.02–0.06	0.02–0.31	0.02–0.27
Cd in control point		0. 01	0.02	0.09	0.06
Cu	Mean	5.11	7.08	7.97	7.81
	Std. deviation	0.68	1.83	3.42	3.80
	Range	4.30–6.33	4.9–10.79	3.12–14.32	2.8–15.84
Cu in control point		4.00	8.00	3.00	2.60
Ni	Mean	2.10	2.17	2.76	2.33
	Std. deviation	0.76	0.58	0.80	0.62
	Range	1.20–3.59	1.30–3.21	1.50–3.98	1.4–3.7
Ni in control point		1.70	2.40	2.10	1.00
Pb	Mean	1.24	1.63	1.55	1.39
	Std. deviation	0.43	0.53	0.70	0.64
	Range	0.45–1.75	0.87–2.93	0.56–3.05	0.67–2.70
Pb in control point		0.70	1.40	0.90	0.80
Zn	Mean	24.82	22.38	22.50	17.77
	Std. deviation	8.28	4.66	11.46	6.95
	Range	43.00–9	32.30–14	45.00–8.8	3.00–31
Zn in control point		9.00	36.00	9.00	13.00
Na %	Mean	0.23	0.28	0.37	0.38
	Std. deviation	0.36	0.30	0.27	0.26
	Range	0.06–1.20	0.05–0.98	0.04–0.80	0.05–0.78
Na in control point		0.05	0.54	0.26	0.24

In recent years, increasing rate of drying in saline Urmia Lake as emerging natural phenomenon, has led to air pollution and raised public health concern. We employed four popular local trees as biomonitors to determine the levels of heavy metals and sodium in their leaves and to evaluate their air pollution tolerance index (APTI). Maximum and minimum mean values of APTI were found for *Vitis vinifera* (12.57 ± 3.83) and *Popolus alba* (6.71 ± 0.99), respectively. The heavy metals concentrations in the tree leaves follow in the order of: Zn > Cu > Ni > Pb > As > Cd. The concentrations of As, Cd and Pb were lower, ranging from 0.03 to 2.93 mg/kg. Due to toxic characteristic, these are not considered as essential micro elements in leaves, but non excessive values of Zn, Cu and Ni are essential for plant growth. Sodium is a vital macro element in tree leaves, so, a maximum range was found in *P. alba* leaves (0.05–0.78%).

According to our findings, the *Vitis vinifera* is suggested to be a suitable local tree for development of greenbelt around Urmia Lake as an air pollutants sink in order to improve air quality of surrounding area, and *P. alba* with lowest APTI is recommended as a sensitive tree for biomonitoring of air pollution. However, there are still needs to supplementary and future researches to find more practical solution for dust elimination originated from the Urmia Lake.

## Declaration of Competing Interest

Authors have no conflict of interests.
